# Supplementation With Phytogenic Compounds Modulates Salivation and Salivary Physico-Chemical Composition in Cattle Fed a High-Concentrate Diet

**DOI:** 10.3389/fphys.2021.645529

**Published:** 2021-06-03

**Authors:** Sara Ricci, Raul Rivera-Chacon, Renee M. Petri, Arife Sener-Aydemir, Suchitra Sharma, Nicole Reisinger, Qendrim Zebeli, Ezequias Castillo-Lopez

**Affiliations:** ^1^Institute of Animal Nutrition and Functional Plant Compounds, Department for Farm Animals and Veterinary Public Health, University of Veterinary Medicine Vienna, Vienna, Austria; ^2^Christian Doppler Laboratory for Innovative Gut Health Concepts of Livestock, Vienna, Austria; ^3^BIOMIN Research Center, BIOMIN Holding GmbH, Tulln, Austria

**Keywords:** saliva composition, salivation, cattle, phytogenic compound, high-concentrate

## Abstract

Saliva facilitates feed ingestion, nutrient circulation, and represents an important pH buffer for ruminants, especially for cattle fed high-concentrate diets that promote rumen acidification. This experiment evaluated the short-term effects of nine phytogenic compounds on salivation, saliva physico-chemical composition as well as ingested feed boli characteristics in cattle. A total of nine ruminally cannulated Holstein cows were used. Each compound was tested in four of these cows as part of a high-concentrate meal (2.5 kg of total mixed ration in dry matter basis for 4 h) in low or high dose, and was compared to a control meal without compound. Saliva was sampled orally (unstimulated saliva) for physico-chemical composition analysis. Composition of the ingested saliva (stimulated saliva), salivation and feed boli characteristics were assessed from ingesta collected at the cardia during the first 30 min of the meal. Analysis of unstimulated saliva showed that supplementation with capsaicin and thyme oil increased buffer capacity, while supplementation with thymol, L-menthol and gentian root decreased saliva pH. In addition, supplementing angelica root decreased saliva osmolality. Regression analysis on unstimulated saliva showed negative associations between mucins and bicarbonate as well as with phosphate when garlic oil, thyme oil or angelica root was supplemented. Analysis of stimulated saliva demonstrated that supplementation with garlic oil increased phosphate concentration, thyme oil tended to increase osmolality, capsaicin and thymol increased buffer capacity, and ginger increased phosphate content. Furthermore, salivation rate increased with ginger and thymol, and tended to increase with garlic oil, capsaicin, L-menthol and mint oil. Feed ensalivation increased with capsaicin. A positive association was found between feed bolus size and salivation rate when any of the phytogenic compounds was supplemented. Overall, our results demonstrate positive short-term effects of several phytogenic compounds on unstimulated and stimulated saliva physico-chemical properties, salivation or feed boli characteristics. Thus, the phytogenic compounds enhancing salivary physico-chemical composition have the potential to contribute to maintain or improve ruminal health in cattle fed concentrate-rich rations.

## Introduction

Salivary secretion is an important body fluid, rich in buffers like bicarbonate, nitrogen compounds and phosphate, and contains several important bioactive compounds, making saliva essential for food perception and ingestion, as well as the oral and gastrointestinal health (Humphrey and Williamson, 2001). Digestive physiology of cattle relies on large amounts of saliva that exceed 150 l/day, which is produced during their long chewing periods (Allen, 1997). Besides being essential for bolus formation, swallowing, nutrient release and circulation, saliva acts in cattle as a buffer that protects the rumen against a drop in pH, which commonly occurs in response to high concentrate feeding (Hofmann, 1989; Allen, 1997). Thus, saliva has a crucial role in rumen function and health in cattle (Fouhse et al., 2017).

Saliva has been extensively studied in humans (Hofman, 2001), and can provide valuable insights as a diagnostic tool in animals as well (Prickett and Zimmerman, 2010). Its composition and the dynamics of secretion have been the target of research for decades in various species (Bailey and Balch, 1961a, b; Carr, 1984). It is well established that diet is the most influencing factor on saliva flow by altering the chewing behavior of cows (Emery et al., 1960; Zebeli et al., 2012; Humer et al., 2018). However, despite its importance for digestion and health, studies evaluating the effect of diet on composition of unstimulated and eating-stimulated salivary secretions are limited in cattle. Research has proven that salivation rate and feed ensalivation are reduced in cattle fed a high-concentrate diet (Chibisa et al., 2016). Additionally, our group recently demonstrated that high-concentrate diet affects ensalivation and the physico-chemical characteristics of saliva including osmolality, mucin, lysozyme activity and main buffers such as bicarbonate and phosphate. These effects were observed either in unstimulated or stimulated saliva with implications for the regulation of ruminal pH of the cows (Castillo-Lopez et al., 2021a).

Apart from mechanical stimuli, research in other species has shown that taste and smell play an important role in the activity of salivary glands (Proctor, 2016). Phytogenic compounds have strong organoleptic properties and have a stimulatory effect on saliva secretion in humans (Nasrawi and Pangborn, 1990; Gardner et al., 2020). In a study evaluating the effects of different phytogenic compounds on chewing activity and ruminal fermentation in dairy cows, we found that certain compounds increased chewing time or influenced rumen fermentation profile, which has the potential to modulate ruminal pH (Castillo-Lopez et al., 2021b). Nonetheless, the effects of these phytogenic compounds on salivation and the physico-chemical properties of saliva in cattle fed high-concentrate diets need to be investigated. Therefore, the aim of the present study was to evaluate the short-term effects of nine phytogenic compounds on saliva composition, salivation and ingested feed boli characteristics in cattle fed a high-concentrate diet. Our hypothesis is that phytogenic compounds can modulate saliva production and physico-chemical composition, thus displaying potential benefits on ruminal health in cattle fed high-concentrate diets.

## Materials and Methods

### Animals and Housing

The methods and protocols followed in this experiment were approved by the Institutional Ethics and Animal Welfare Committee of the University of Veterinary Medicine Vienna and the Austrian national authority according to the law for animal experiments (protocol number: BMNWF- 68.205/0003-V/3b/2019).

This study was part of a larger research elucidating the role of phytogenic compounds on chewing and salivary composition as well as rumen health in cattle fed concentrate-rich diets. Results of feed intake, chewing and rumen variables have been reported elsewhere (Castillo-Lopez et al., 2021b). In this trial, nine ruminally cannulated, non-lactating Holstein cows (887 ± 72.4 kg) were group-housed at the research dairy farm of the University of Veterinary Medicine, Vienna. Before the start of the experiment, cows were fed a forage diet and then transitioned during 1 week to a high concentrate diet containing on dry matter (DM) basis 26.25% grass silage, 8.75% corn silage, 26% rolled wheat, and 39% pelleted concentrate mixture. The diet had 32.8% starch, 41.9% non-fiber carbohydrates and 12.9% physically effective Neutral Detergent Fiber (NDF) (peNDF > 8 mm) (Supplementary Table 1), a diet considered with high acidogenic potential in cattle (Khorrami et al., 2021). All nutrients requirements for adult dairy cattle were met or exceeded. For sampling and animal management purposes, cows were blocked based on body weight in two groups with 5 and 4 cows, respectively. Cows were housed in a free-stall barn with individual deep cubicles (2.6 × 1.25 m) and straw bedding. Free choice mineral blocks and water were available *ad libitum*, except during the treatment meal consumption. Diet was mixed once a day using an automated feeding system (Trioliet Triomatic T15, Oldenzaal, Netherlands), and offered to cows as total mixed ration (TMR) in individual feed bunks equipped with electronic weigh scales (Insentec B.V., Marknesse, Netherlands). In order to increase palatability and reduce sorting, water was added to the TMR during mixing to target 46% DM content.

### Treatments and Experimental Design

Details on phytogenic compound production and extraction as well as the strategy followed for the estimation of minimum number of animals needed for adequate statistical power have been reported in our companion paper (Castillo-Lopez et al., 2021b). Briefly, the nine phytogenic compounds evaluated were angelica root, capsaicin, garlic oil, gentian root, ginger, L-menthol, mint oil, thyme oil, and thymol (Supplementary Table 2). The compounds were in powder form and were mixed in either low (1×, LOW) or high dosage (10×, HIGH) with 50 g of a carrier consisting of silica wheat. This mixture was then combined with 2.5 kg of TMR (DM basis). The control meal (CON) was prepared by combining 50 mg of the carrier with 2.5 kg of TMR. Treatments and control were offered in the feed bunks and cows were allowed to eat during a 4-h meal, then orts were weighed and discarded. The experimental design included 4 cows per treatment, meaning that each substance at each dosage was tested on four of the nine cows, using an incomplete Latin Square experimental design. Short-term effects were investigated, so that response variables for each evaluation were assessed on a single day, with four compounds tested per experimental day. Compounds were randomly allocated to the cows, so that each animal consumed both dosages of the corresponding phytogenic compound, and the treatment sequence was balanced for carry-over effects. The two dosages of each compound were tested in different days, with the LOW dosage being tested first. After each treatment assessment, there was a washout day in which cows consumed the CON diet. Each cow served as its own control, and the control measurements were performed at the beginning and at the end of the experiment, when the animals received the CON diet. Before offering the 4-h treatment meal, feed was automatically restricted for 9 h in order to promote feed consumption.

### Feed Chemical Analyses

Feed samples were collected daily and pooled by week for chemical composition analyses. After drying samples at 65°C in a forced-air oven for 48 h, they were ground through a 0.5 mm screen (Ultra Centrifugal Mill ZM 200, Retsch, Haan, Germany). Ash was analyzed by combustion overnight at 580°C. Crude protein (CP) was analyzed with the Kjeldahl method (VDLUFA, 2012) and ether extract with the soxhlet system (Extraction System B-811, Büchi, Flawil, Switzerland). NDF and Acid Detergent Fiber (ADF) contents were determined with sodium sulfite following the official analytical methods of VDLUFA (2012), and using the Fiber Therm FT 12 (Gerhardt GmbH and Co., KG, Königswinter, Germany) with heat-stable α-amylase for NDF. Starch content was measured with the K-TSTA kit (Megazyme Ltd.). Non-fiber carbohydrates were calculated as 100 – (% CP + %NDF + % ether extract + % ash).

### Saliva Sampling and Analysis

#### Unstimulated Saliva Collection

**FIGURE 1:**
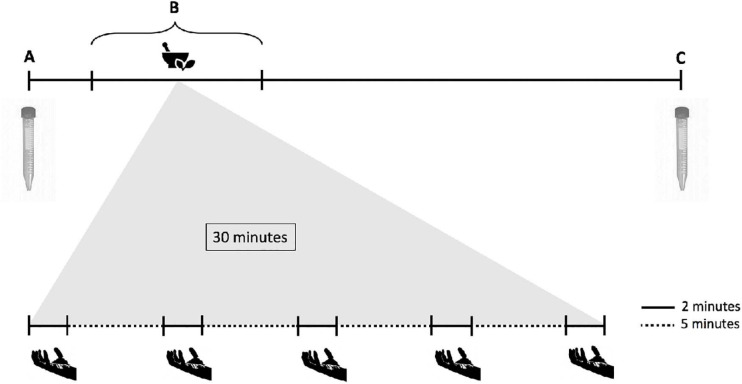
Schematic illustration of the sampling procedure timeline. **(A)** Unstimulated saliva was collected from the mouth, then the rumen was partially emptied. **(B)** The cows were offered the controlled meal with phytogenic compounds or control. Feed boli sampling from the cardia (collection of stimulated saliva) was performed over the first 30 min of the meal, with 2 min for collection alternated with 5 min of rest. **(C)** Unstimulated saliva sampling, and end of the 4-h meal.

Cows were tied using a halter and unstimulated saliva was sampled directly from the mouth (in the space between the teeth and the cheek) using a vacuum-pump with a maximum suction power of – 80 kPa (model Kataspir 30, MEDUTEK, GmbH and Co., KG., Bremen, Germany). Saliva was collected immediately before and 4 h after offering the meal (Figure 1). Approximately 100 mL of saliva were collected at each sampling time, divided in ten aliquots, and stored in 15 mL vials. Saliva pH was measured immediately after sampling, with a portable pH meter (Mettler-Toledo, AG; Analytical CH; Schwerzenbach, Switzerland), and the samples were frozen at −20°C for further analysis. The saliva container of the pump was washed and dried between collections.

#### Stimulated Saliva Collection and Measurement of Salivation

Ingested feed boli collections were conducted following the protocols of Maekawa et al. (2002), with minor modifications. First, the rumen was partially emptied and the digesta was stored in an insulated and pre-warmed 50 L polyethylene barrel, in order to keep it warm. Once the anterior pillar of the rumen was exposed and the cardia could be reached without interference of reticular digesta, the meal with either phytogenic treatment or control was offered. Then, swallowed feed boli were collected from the cardia while the cows were eating by inserting an arm through the ruminal cannula and using a plastic bag (17 × 30 cm) secured on the wrist with a rubber band. Up to five feed boli were collected from each cow over a 30-min time frame (2 min of sampling, followed by 5 min of break during which cows were left undisturbed) (Figure 1). Each sampled bolus was weighed and then strained using four layers of gauze to collect stimulated saliva. The fluid was collected in a 50 mL vial and pH was measured. At the end of the sampling procedure, ruminal content was returned into the rumen. Saliva and feed boli were then frozen at −20°C for further analysis. Saliva content of feed boli was calculated as the difference in moisture content between the feed offered and the collected ingesta samples. The DM content of feed boli were determined by oven drying at 55°C for 72 h. Feed ensalivation was calculated as the amount of saliva added to each gram of feed ingested (DM basis). Salivation rate (g/min) calculations were conducted by dividing the content of saliva from each bolus by collection time (2 min). In addition, feed ensalivation per unit of metabolic weight of cows (live weight)^0.75^ was calculated. Conversion of saliva weight to volume was performed according to Maekawa et al. (2002) assuming that 1 g of saliva equals 1 mL, because the DM of saliva has been shown to be minimal (Bailey and Balch, 1961a).

#### Composition Analyses for Unstimulated and Stimulated Saliva

The parameters evaluated to assess unstimulated saliva composition were pH, bicarbonate concentration, phosphate concentration, total proteins, mucin concentration, buffer capacity, osmolality, and lysozyme activity. Saliva pH was measured using a manual pH meter (Mettler-Toledo, AG; Analytical CH; Schwerzenbach, Switzerland). Bicarbonate concentration was estimated according to an enzymatic colorimetric assay (Zabokova-Bilbilova et al., 2013) and following the Diagnostic Systems Kit (DiaSys GmbH, # 109509910021, Holzheim, Germany). Phosphate concentration was assessed with a colorimetric method (Fiyaz et al., 2013) using the Malachite Green Phosphate Assay Kit (MAK307, Sigma-Aldrich, Austria). Total proteins were measured according to Bradford (1976), also used by Roa et al. (2008) and Kejriwal et al. (2015) with minor modifications. Mucin concentration was measured according to the protocol described by Kilcoyne et al. (2011) with minor modifications. Buffer capacity was measured according to the method utilized by Pinto Antunes et al. (2015). Osmolality was determined according to the method used by Villiger et al. (2018) with minor modifications. Lysozyme activity was determined according to the procedure used by Helal and Melzig (2008) and following the Lysozyme Detection Kit (Sigma-Aldrich, # LY0100, Austria) with minor modifications. For stimulated saliva, samples were thawed on ice, centrifuged at 4°C for 20 min at 15,000 rpm and aliquots were prepared to measure pH, phosphate concentration, buffer capacity, and osmolality. Due to the dark appearance of the stimulated saliva, it was not possible to measure bicarbonate concentration, total proteins, mucin concentration, and lysozyme activity in these samples. Minor modifications for the listed protocols used are described in Castillo-Lopez et al. (2021a).

### Statistical Analysis

Data were analyzed with the Proc Mixed of SAS (version 9.4; SAS Institute, Cary, NC, United States) with treatment dose as fixed effects and cow within experimental block as random effect, linear and quadratic effects were evaluated. Data on unstimulated saliva composition prior to treatment or control meal intake were used as covariate measurements. Data of feed boli from the same cow within treatment dose or control in different times were processed as repeated measures in the analysis with a first order variance-covariance structure matrix taking into consideration that the covariance decays with time. Data were also checked for normal distribution using Proc Univariate followed by the normal and plot options. The largest standard error of the mean (SEM) is reported. Statistical significance was declared when *P* ≤ 0.05 and tendency was mentioned if 0.05 < *P* ≤ 0.10. Correlation heatmaps were constructed with (R Core Team., 2020) using the corrplot package (Wei and Simko, 2017). Then, based on results from correlation analysis, linear regressions were performed with SAS to evaluate the association among salivary components, salivation dynamics and chewing index. Additionally, *a posteriori* statistical power analysis was conducted according to Stroup (1999) and Kononoff and Hanford (2006), which displayed an acceptable statistical power with an average of 0.812 with α = 0.05.

## Results

### Unstimulated Saliva Composition

**FIGURE 2:**
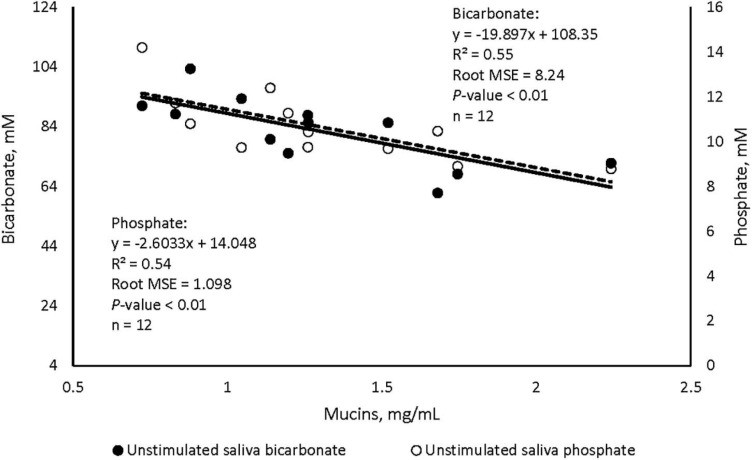
Regression plots illustrating the associations between mucin concentration and bicarbonate (⚫), and phosphate (-○-) in the unstimulated salivary secretions of cattle, plots include average values across angelica root, garlic oil, and thyme oil.

Data listed in Table 1 indicate that supplementing garlic oil tended to linearly enhance bicarbonate concentration and the buffer capacity of saliva (*P* = 0.06), while the low dose tended to reduce total proteins (*P* = 0.09). Mucins concentration tended to decrease after cows received the meal with garlic oil (*P* = 0.06). Thyme oil linearly increased the buffer capacity (*P* < 0.05) and tended to quadratically affect (*P* = 0.10) mucin concentration (Table 2). The regression analysis revealed negative associations between mucin concentration and bicarbonate as well as phosphate in the unstimulated salivary secretions when garlic oil, thyme oil or angelica root were supplemented (Figure 2). Accordingly, for every mg/mL increment in mucin concentration, bicarbonate and phosphate concentration decreased by 19.9 and 2.6 mM, respectively. Additionally, the unstimulated saliva pH generally dropped after supplementation with five phytogenic compounds, especially when using a high dosage. For example, the high dosages of thyme oil and capsaicin tended (*P* = 0.09) to lower salivary pH (Tables 2, 3), while thymol (*P* < 0.05) and L-menthol (*P* < 0.05) significantly decreased this variable (Tables 4, 5). Supplementation with angelica root showed a quadratic effect on saliva osmolality (*P* < 0.01) (Supplementary Table 3). Gentian root decreased salivary pH (*P* < 0.05) (Supplementary Table 4). Mint oil and ginger did not affect any of the unstimulated saliva composition parameters.

**TABLE 1:** Effect of supplementation with garlic oil on salivary physico-chemical properties, salivation, and feed bolus dynamics of non-lactating Holstein dairy cows.

	**Treatment^1^**			***P*-value**		
**Unstimulated saliva^2^**	**CON**	**LOW**	**HIGH**	**SEM^4^**	**Linear**	**Quadratic**
pH	8.82	8.78	8.73	0.062	0.25	0.99
Bicarbonate, mM	71.26	93.34	95.16	8.620	0.06	0.32
Phosphate, mM	9.71	10.88	11.76	1.100	0.15	0.91
Total proteins, μg/mL	380.9	184.7	312.9	71.81	0.47	0.09
Buffer capacity, mol of HCl/L/ΔpH	0.014	0.016	0.018	0.0015	0.06	0.82
Osmolality, mOsm/kg	246.8	251.7	251.3	10.33	0.73	0.83
Lysozyme activity, U/mL/min	33.88	36.93	43.56	6.350	0.23	0.82
Mucins, mg/mL	1.56	1.01	0.93	0.195	0.06	0.38
**Stimulated saliva^3^**						
pH	6.79	6.55	6.71	0.0709	0.25	0.01
Phosphate, mM	12.52	17.35	14.84	1.287	0.03	<0.01
Buffer capacity, mol of HCl/L/ΔpH	0.035	0.026	0.036	0.0034	0.66	0.01
Osmolality, mOsmol/kg	402.45	546.81	437.79	66.009	0.38	<0.01
**Saliva dynamics**						
Salivation rate, g/min	67.40	83.24	79.81	5.578	0.06	0.14
Ensalivation, g/g DM feed	5.05	3.62	4.39	0.509	0.33	0.13
Ensalivation, l/kg DM feed/kg LW^0.75^	0.033	0.023	0.028	0.004	0.21	0.09
Bolus size (as is), g	213.29	272.78	284.77	21.475	0.12	0.08
Bolus size (DM), g	34.24	49.78	41.58	5.573	0.20	0.05

*^1^CON: a control diet prepared by combining 50 g of the wheat silica carrier and 2.5 kg (DM) of TMR; LOW: 0.3 ppm of garlic oil in 2.5 kg (DM) of TMR; HIGH: 3 ppm of garlic oil in 2.5 kg (DM) of TMR.^2^Saliva samples collected from the mouth after treatment intake; data from samples collected before treatment were used as covariates.^3^Saliva samples collected at cardia by collecting and straining saliva from ingested feed boli.^4^The largest standard error of the mean.*

**TABLE 2:** Effect of supplementation with thyme oil on salivary physico-chemical properties, salivation, and feed bolus dynamics of non-lactating Holstein dairy cows.

	**Treatment^1^**			***P*-value**		
**Unstimulated saliva^2^**	**CON**	**LOW**	**HIGH**	**SEM^4^**	**Linear**	**Quadratic**
pH	8.81	8.81	8.70	0.0520	0.09	0.37
Bicarbonate, mM	76.71	77.61	83.76	10.734	0.56	0.82
Phosphate, mM	10.45	9.32	10.12	1.532	0.87	0.59
Total proteins, μg/mL	352.7	200.6	397.2	93.87	0.69	0.15
Buffer capacity, mol of HCl/L/ΔpH	0.013	0.015	0.018	0.0013	0.01	0.97
Osmolality, mOsm/kg	243.5	238.6	261.4	12.81	0.17	0.25
Lysozyme activity, U/mL/min	31.27	42.35	34.15	5.539	0.63	0.13
Mucins, mg/mL	1.53	0.95	1.26	0.193	0.36	0.10
**Stimulated saliva^3^**						
pH	6.86	6.63	6.89	0.131	0.66	<0.01
Phosphate, mM	11.67	13.49	11.66	1.533	0.99	0.06
Buffer capacity, mol of HCl/L/ΔpH	0.029	0.022	0.025	0.0038	0.32	0.14
Osmolality, mOsmol/kg	409.69	533.76	488.76	53.521	0.09	0.07
**Saliva dynamics**						
Salivation rate, g/min	60.15	80.40	73.75	12.310	0.23	0.17
Ensalivation, g/g DM feed	4.61	4.47	5.99	1.261	0.07	0.20
Ensalivation, l/kg DM feed/kg LW^0.75^	0.719	0.954	0.887	0.139	0.21	0.20
Bolus size (as is), g	195.90	246.94	239.54	38.867	0.12	0.22
Bolus size (DM), g	29.46	42.63	41.47	9.623	0.03	0.14

*^1^CON: a control diet prepared by combining 50 g of the wheat silica carrier and 2.5 kg (DM) of TMR; LOW: 9.4 ppm of thyme oil in 2.5 kg (DM) of TMR; HIGH: 94 ppm of thyme oil in 2.5 kg (DM) of TMR.^2^Saliva samples collected from the mouth after treatment intake; data from samples collected before treatment were used as covariates.^3^Saliva samples collected at cardia by collecting and straining saliva from ingested feed boli.^4^The largest standard error of the mean.*

**TABLE 3:** Effect of supplementation with capsaicin on salivary physico-chemical properties, salivation, and feed bolus dynamics of non-lactating Holstein dairy cows.

	**Treatment^1^**			***P*-value**		
**Unstimulated saliva^2^**	**CON**	**LOW**	**HIGH**	**SEM^4^**	**Linear**	**Quadratic**
pH	8.83	8.84	8.75	0.047	0.09	0.22
Bicarbonate, mM	74.12	81.17	75.52	5.758	0.84	0.37
Phosphate, mM	11.15	11.98	14.08	1.756	0.23	0.74
Total proteins, μg/mL	275.1	267.7	263.6	128.84	0.93	1.00
Buffer capacity, mol of HCl/L/ΔpH	0.013	0.015	0.014	0.0014	0.58	0.50
Osmolality, mOsm/kg	242.2	247.8	231.2	11.42	0.31	0.30
Lysozyme activity, U/mL/min	34.11	38.95	26.51	7.916	0.45	0.39
Mucins, mg/mL	1.52	1.64	1.48	0.465	0.94	0.81
**Stimulated saliva^3^**						
pH	6.97	6.71	6.90	0.158	0.58	0.09
Phosphate, mM	10.25	11.18	12.62	1.408	0.07	0.86
Buffer capacity, mol of HCl/L/ΔpH	0.036	0.050	0.118	0.0447	0.03	0.56
Osmolality, mOsmol/kg	365.91	440.62	312.59	81.004	0.44	0.13
**Saliva dynamics**						
Salivation rate, g/min	69.43	92.79	56.27	10.778	0.06	<0.01
Ensalivation, g/g DM feed	5.87	4.00	8.02	1.562	0.03	0.01
Ensalivation, l/kg DM feed/kg LW^0.75^	0.036	0.024	0.049	0.010	0.03	0.02
Bolus size (as is), g	203.25	293.42	149.78	39.367	0.02	<0.01
Bolus size (DM), g	29.94	50.04	17.37	8.947	<0.01	<0.01

*^1^CON: a control diet prepared by combining 50 g of the wheat silica carrier and 2.5 kg (DM) of TMR; LOW: 10 ppm of capsaicin in 2.5 kg (DM) of TMR; HIGH: 100 ppm of capsaicin in 2.5 kg (DM) of TMR.^2^Saliva samples collected from the mouth after treatment intake; data from samples collected before treatment were used as covariates.^3^Saliva samples collected at cardia by collecting and straining saliva from ingested feed boli.^4^The largest standard error of the mean.*

**TABLE 4:** Effect of supplementation with thymol on salivary physico-chemical properties, salivation, and feed bolus dynamics of non-lactating Holstein dairy cows.

	**Treatment^1^**			***P*-value**		
**Unstimulated saliva^2^**	**CON**	**LOW**	**HIGH**	**SEM^4^**	**Linear**	**Quadratic**
pH	8.86	8.80	8.71	0.052	0.04	0.82
Bicarbonate, mM	81.81	83.99	88.22	7.412	0.34	0.86
Phosphate, mM	9.85	9.71	12.53	1.735	0.22	0.42
Total proteins, μg/mL	362.0	295.5	309.2	93.45	0.70	0.72
Buffer capacity, mol of HCl/L/ΔpH	0.014	0.017	0.016	0.0014	0.31	0.22
Osmolality, mOsm/kg	253.1	244.5	253.5	6.35	0.97	0.22
Lysozyme activity, U/mL/min	39.56	34.68	38.05	8.833	0.88	0.63
Mucins, mg/mL	1.52	1.38	1.52	0.503	0.99	0.80
**Stimulated saliva^3^**						
pH	6.83	6.44	6.72	0.099	0.31	<0.01
Phosphate, mM	12.11	14.49	12.00	2.336	0.95	0.13
Buffer capacity, mol of HCl/L/ΔpH	0.041	0.056	0.133	0.0335	0.01	0.24
Osmolality, mOsmol/kg	377.58	413.25	379.95	68.880	0.95	0.35
**Saliva dynamics**						
Salivation rate, g/min	71.25	71.81	96.33	8.996	0.04	0.24
Ensalivation, g/g DM feed	4.96	4.54	3.97	0.857	0.35	0.95
Ensalivation, l/kg DM feed/kg LW^0.75^	0.032	0.028	0.025	0.001	0.25	0.96
Bolus size (as is), g	226.61	231.40	309.02	35.781	0.04	0.29
Bolus size (DM), g	36.44	40.55	53.33	9.025	0.06	0.58

*^1^CON: a control diet prepared by combining 50 g of the wheat silica carrier and 2.5 kg (DM) of TMR; LOW: 5 ppm of thymol in 2.5 kg (DM) of TMR; HIGH: 50 ppm of thymol in 2.5 kg (DM) of TMR.^2^Saliva samples collected from the mouth after treatment intake; data from samples collected before treatment were used as covariates.^3^Saliva samples collected at cardia by collecting and straining saliva from ingested feed boli.^4^The largest standard error of the mean.*

**TABLE 5:** Effect of supplementation with L-menthol on salivary physico-chemical properties, salivation, and feed bolus dynamics of non-lactating Holstein dairy cows.

	**Treatment^1^**			***P*-value**		
**Unstimulated saliva^2^**	**CON**	**LOW**	**HIGH**	**SEM^4^**	**Linear**	**Quadratic**
pH	8.78	8.76	8.59	0.067	0.01	0.25
Bicarbonate, mM	77.82	76.04	85.20	8.960	0.42	0.52
Phosphate, mM	10.99	9.83	11.11	1.285	0.94	0.40
Total proteins, μg/mL	353.2	374.5	449.1	131.27	0.55	0.87
Buffer capacity, mol of HCl/L/ΔpH	0.013	0.014	0.016	0.0014	0.15	0.82
Osmolality, mOsm/kg	244.5	245.0	241.7	12.62	0.86	0.90
Lysozyme activity, U/mL/min	31.38	36.26	27.45	6.428	0.59	0.34
Mucins, mg/mL	1.39	1.46	1.91	0.352	0.33	0.67
**Stimulated saliva^3^**						
pH	6.88	6.76	6.80	0.103	0.15	0.13
Phosphate, mM	11.73	11.88	12.57	1.374	0.47	0.80
Buffer capacity, mol of HCl/L/ΔpH	0.033	0.055	0.033	0.0120	0.97	0.09
Osmolality, mOsmol/kg	381.88	432.18	427.36	46.839	0.25	0.44
**Saliva dynamics**						
Salivation rate, g/min	61.14	72.65	72.98	8.508	0.08	0.29
Ensalivation, g/g DM feed	6.13	6.92	5.61	1.900	0.64	0.30
Ensalivation, l/kg DM feed/kg LW^0.75^	0.037	0.042	0.035	0.011	0.70	0.31
Bolus size (as is), g	177.75	216.51	218.23	34.506	0.06	0.33
Bolus size (DM), g	25.58	33.67	33.47	7.963	0.07	0.29

*^1^CON: a control diet prepared by combining 50 g of the wheat silica carrier and 2.5 kg (DM) of TMR; LOW: 6.7 ppm of L-menthol in 2.5 kg (DM) of TMR; HIGH: 67 ppm of L-menthol in 2.5 kg (DM) of TMR.^2^Saliva samples collected from the mouth after treatment intake; data from samples collected before treatment were used as covariates.^3^Saliva samples collected at cardia by collecting and straining saliva from ingested feed boli.^4^The largest standard error of the mean.*

### Stimulated Saliva Composition

**FIGURE 3:**
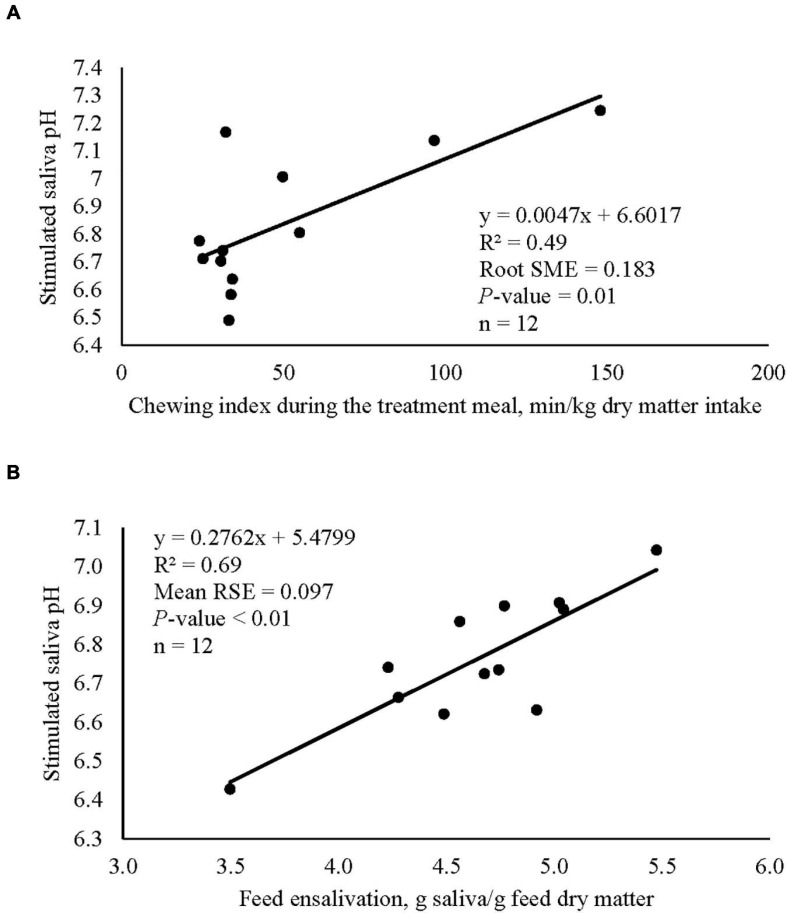
Regression plots showing the association between stimulated salivary pH of cows and chewing index during the meal **(A)**, plot includes the averaged values of capsaicin and thyme oil, and the association between stimulated salivary pH and feed ensalivation **(B)**, plot includes the averaged values of capsaicin, garlic oil, gentian root, and mint oil.

Low dose of garlic oil increased stimulated saliva osmolality and phosphate (*P* < 0.01), but reduced pH and buffer capacity (*P* < 0.05) (Table 1). Low dose of thyme oil decreased pH (*P* < 0.01) and tended to increase phosphate concentration (*P* = 0.06) and osmolality (*P* = 0.07) (Table 2). Low dose of thymol decreased pH (*P* < 0.01), but this compound linearly increased buffer capacity (*P* < 0.05) (Table 4). The low dose of capsaicin showed a trend to reduce pH (*P* = 0.09) and a significant linear increase of buffer capacity (*P* < 0.05) (Table 3). Supplementation with ginger reduced salivary pH (*P* < 0.01), but increased phosphate concentration (*P* < 0.01) (Table 6). Low dosage of L-menthol showed a trend to increase buffer capacity (*P* = 0.09) (Table 5). Low dose of mint oil decreased pH (*P* < 0.05), but increased salivary phosphate concentration (*P* < 0.01). However, mint oil tended to linearly decrease osmolality (*P* = 0.09) (Table 7). Supplementing angelica root tended to quadratically decrease pH (*P* = 0.09) and osmolality (*P* = 0.07) (Supplementary Table 3). Low dose of gentian root reduced salivary pH (*P* < 0.05), but increased phosphate concentration (*P* < 0.05) and the buffer capacity (*P* = 0.05) (Supplementary Table 4). Regression analysis showed that for every unit increment in chewing index during the meal, stimulated saliva pH increased by 0.005 when capsaicin or thyme oil were supplemented (Figure 3A). Additionally, a positive association was found between stimulated saliva pH and feed ensalivation when supplementing garlic oil, capsaicin, mint oil or gentian root. As shown in the regression plot, for every gram increment in feed ensalivation, salivary pH increased by 0.28 units (Figure 3B).

**TABLE 6:** Effect of supplementation with ginger on salivary physico-chemical properties, and feed bolus dynamics of non-lactating Holstein dairy cows.

	**Treatment^1^**			***P*-value**		
**Unstimulated saliva^2^**	**CON**	**LOW**	**HIGH**	**SEM^4^**	**Linear**	**Quadratic**
pH	8.77	8.72	8.71	0.061	0.48	0.73
Bicarbonate, mM	82.53	82.82	84.81	11.055	0.87	0.95
Phosphate, mM	10.33	10.14	11.15	0.909	0.46	0.57
Total proteins, μg/mL	348.5	316.5	396.4	111.47	0.75	0.66
Buffer capacity, mol of HCl/L/ΔpH	0.014	0.016	0.014	0.0011	0.84	0.15
Osmolality, mOsm/kg	248.7	257.2	246.0	11.18	0.84	0.46
Lysozyme activity, U/mL/min	33.70	37.28	31.08	4.783	0.66	0.39
Mucins, mg/mL	1.04	1.13	1.18	0.288	0.74	0.95
**Stimulated saliva^3^**						
pH	6.76	6.61	6.58	0.056	<0.01	0.34
Phosphate, mM	12.99	14.94	17.73	1.044	<0.01	0.71
Buffer capacity, mol of HCl/L/ΔpH	0.038	0.033	0.032	0.0041	0.24	0.75
Osmolality, mOsmol/kg	368.25	452.58	433.35	55.700	0.13	0.22
**Saliva dynamics**						
Salivation rate, g/min	68.95	86.35	93.15	6.520	0.01	0.46
Ensalivation, g/g DM feed	4.33	4.04	3.86	0.469	0.32	0.91
Ensalivation, l/kg DM feed/kg LW^0.75^	0.027	0.025	0.024	0.003	0.29	0.94
Bolus size (as is), g	214.24	272.47	292.18	25.487	<0.01	0.50
Bolus size (DM), g	35.37	45.65	49.01	6.829	0.05	0.65

*^1^CON: a control diet prepared by combining 50 g of the wheat silica carrier and 2.5 kg (DM) of TMR; LOW: 40 ppm of ginger in 2.5 kg (DM) of TMR; HIGH: 400 ppm of ginger in 2.5 kg (DM) of TMR.^2^Saliva samples collected from the mouth after treatment intake; data from samples collected before treatment were used as covariates.^3^Saliva samples collected at cardia by collecting and straining saliva from ingested feed boli.^4^The largest standard error of the mean.*

**TABLE 7:** Effect of supplementation with mint oil on salivary physico-chemical properties, salivation, and feed bolus dynamics of non-lactating Holstein dairy cows.

	**Treatment^1^**			***P*-value**		
**Unstimulated saliva^2^**	**CON**	**LOW**	**HIGH**	**SEM^4^**	**Linear**	**Quadratic**
pH	8.85	8.81	8.75	0.064	0.15	0.80
Bicarbonate, mM	82.03	71.28	82.21	11.714	0.99	0.38
Phosphate, mM	11.82	9.25	7.64	1.893	0.15	0.80
Total proteins, μg/mL	384.1	434.9	518.0	97.00	0.28	0.89
Buffer capacity, mol of HCl/L/ΔpH	0.013	0.013	0.014	0.0011	0.61	0.73
Osmolality, mOsm/kg	241.6	224.4	236.1	16.39	0.77	0.44
Lysozyme activity, U/mL/min	30.39	37.56	50.06	13.757	0.20	0.87
Mucins, mg/mL	1.28	1.51	2.69	0.595	0.17	0.55
**Stimulated saliva^3^**						
pH	6.95	6.57	6.79	0.142	0.20	0.02
Phosphate, mM	11.13	16.24	9.97	2.325	0.55	<0.01
Buffer capacity, mol of HCl/L/ΔpH	0.033	0.030	0.030	0.0096	0.78	0.92
Osmolality, mOsmol/kg	373.74	316.92	301.92	69.190	0.09	0.62
**Saliva dynamics**						
Salivation rate, g/min	68.62	86.39	91.54	13.396	0.08	0.64
Ensalivation, g/g DM feed	5.37	4.17	5.58	1.058	0.69	0.05
Ensalivation, l/kg DM feed/kg LW^0.75^	0.032	0.025	0.034	0.007	0.63	0.02
Bolus size (as is), g	197.31	290.43	273.73	40.663	0.05	0.16
Bolus size (DM), g	28.57	52.20	39.67	8.976	0.12	0.02

*^1^CON: a control diet prepared by combining 50 g of the wheat silica carrier and 2.5 kg (DM) of TMR; LOW: 15.3 ppm of mint oil in 2.5 kg (DM) of TMR; HIGH: 153 ppm of mint oil in 2.5 kg (DM) of TMR.^2^Saliva samples collected from the mouth after treatment intake; data from samples collected before treatment were used as covariates.^3^Saliva samples collected at cardia by collecting and straining saliva from ingested feed boli.^4^The largest standard error of the mean.*

### Salivation and Feed Bolus Dynamics

**FIGURE 4:**
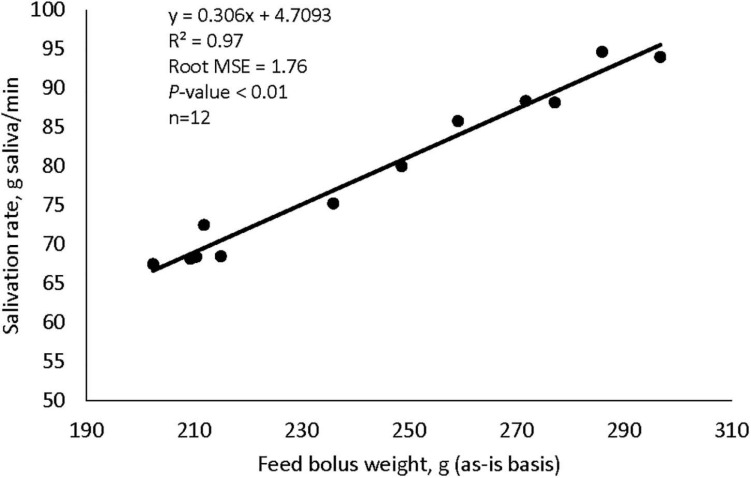
The influence of feed bolus weight on the salivation rate in cattle fed concentrate-rich diets, plot includes values across all nine phytogenic compounds.

Supplementation with garlic oil tended to increase salivation rate (*P* = 0.06), and the low dose increased (*P* = 0.05) bolus size; garlic oil also tended (*P* = 0.09) to display a quadratic effect on feed ensalivation per unit of metabolic body weight (Table 1). Supplementation with thyme oil showed a trend toward a linear increment of feed ensalivation (*P* = 0.07), while causing an increment (*P* < 0.05) of feed boli size on a DM basis (Table 2). Thymol increased salivation rate (*P* < 0.05) and feed boli weight as is (*P* < 0.05) (Table 4). Capsaicin had a strong influence on saliva dynamics. Interestingly, the low dose of capsaicin increased both saliva flow to the rumen (*P* < 0.01) and the bolus size (*P* < 0.01). On the contrary, the high dosage of capsaicin reduced the weight of the feed boli and the flow of saliva to the rumen, while increasing (*P* < 0.05) feed ensalivation. Capsaicin also had a significant quadratic effect on feed ensalivation per unit of metabolic body weight (Table 3). Ginger increased salivation rate (*P* < 0.05) and feed bolus size, both as is (*P* < 0.01) and on DM basis (*P* = 0.05) (Table 6). L-menthol showed a trend toward an increment of boli size (*P* = 0.07), and tended to linearly increase (*P* = 0.08) salivation rate (Table 5). Mint oil tended to increase salivation rate (*P* = 0.08), and its low dosage reduced feed ensalivation (*P* < 0.05), likely due to the increase (*P* < 0.05) in feed bolus size with this dosage; this compound also displayed a quadratic effect on feed ensalivation per unit of metabolic body weight (Table 7). Gentian root tended to increase salivation rate (*P* = 0.08) (Supplementary Table 4). The relationship between the bolus size and salivation rate across all phytogenic compounds is shown in Figure 4. The regression revealed a strong positive effect of bolus size on the salivation rate (*R*^2^ = 0.97). For every gram increment in feed boli weight, salivation rate increased by 0.31 g/min.

## Discussion

Salivation and saliva composition in cattle have received very little attention, despite their importance for digestion and rumen health. Saliva is the result of continuous secretion and mixing of parotid, mandibular, sublingual, and ventral buccal salivary glands (Kay, 1960; Hofmann, 1989) and its secretion depends on many factors, such as gland type, stimuli, activity of the nervous system, individual animal variation, diet composition and organoleptic properties of the feed (Emery et al., 1960; Larsen et al., 1999; Nieuw Amerongen et al., 2007; Thomas et al., 2009). In agreement with our hypothesis, the present study demonstrated that phytogenic compounds are able to positively modulate salivation and saliva composition. Although we did not investigate the mode of action of the substances, we speculate that they exerted organoleptic effects or targeted stimuli resulting in enhancement of salivation or changes of salivary physico-chemical properties. It is also important to note that this experiment included dry cows, with dry matter intake and metabolic activity likely being different compared to lactating cows. However, findings from this experiment contribute to improve the current knowledge on salivation dynamics affected by concentrate-rich diets and may serve as a foundation toward further investigation in lactating cows under similar intensive feeding systems.

Salivary buffer capacity and buffer content are important properties contributing to rumen health in cattle (Bailey and Balch, 1961a), which become even more crucial when the amount of concentrates in the diet is high, as in our study. Findings from this trial show that these properties were enhanced by several phytogenic compounds; in particular, capsaicin increased buffer capacity and tended to increase phosphate content of stimulated saliva. Interestingly, capsaicin increased eating time and its low dose increased rumination time, as shown in the companion study (Castillo-Lopez et al., 2021b), indicating the positive influence of chewing activity for the enhancement of salivary properties in cattle fed high-concentrate diets. In addition, feeding garlic oil had a positive influence on buffer capacity of unstimulated saliva, bicarbonate content, and salivation. These changes may have contributed to lower the concentration of reticular volatile fatty acids, and to enhance ruminal pH post consumption (Castillo-Lopez et al., 2021b). However, we found that higher concentrations of bicarbonate and phosphate were associated with lower content of mucins in unstimulated saliva, and that mucins tended to decrease with garlic oil supplementation. Mucins contribute to dental health and give saliva its viscosity and elasticity to maintain the structure of feed boli (Nieuw Amerongen et al., 2007; Carpenter, 2013). The reduction of these proteins with garlic oil supplementation could be due to the sulfur-based molecules in allicin, one of the components of garlic, which can influence mucin-secreting cells and the proteins structure (Choong et al., 2004; Kudva et al., 2012; Dewi et al., 2017). Nevertheless, further research on the association between salivary buffers, mucin content and their impact on rumen function is needed.

Our results showed that high dose of thyme oil tended to decrease the pH of unstimulated saliva, and the low dose reduced stimulated saliva pH. Thyme oil, and in particular some of its components, such as carvacrol, have been demonstrated to have antimicrobial properties. For instance, Juven et al. (1994) reported the antimicrobial activity to be enhanced in anaerobic conditions and at pH as low as 5.5. Thymol, which is a component of thyme oil, showed a similar pattern and decreased both stimulated and unstimulated saliva pH. This compound has also been shown to have antimicrobial activity, especially in anaerobic conditions and lower pH (Juven et al., 1994; Evans and Martin, 2000; Nagoor Meeran et al., 2017). Thus, the reduction of saliva pH may have beneficial effect for the reinforcement of the antimicrobial activity of these components when fed to cattle. Other compounds that tended or significantly decreased unstimulated salivary pH were L-menthol, capsaicin and gentian root. This change in pH may confer additional benefits to saliva. In fact, saliva contains specific proteins, e.g., proline-rich proteins, amylases, histatins, and cystatins, that interact with plant polyphenols, such as tannins (Hofmann, 1989; Bennick, 2002; Lamy et al., 2011). These potentially harmful compounds are found in different plants, and can be bound only at certain pH levels (Bennick, 2002). Furthermore, Friedman and Jürgens (2000) demonstrated that some polyphenols have their structure irreversibly altered by variations in pH. Therefore, the phytogenic compounds in this study could have triggered a defensive reaction resulting in lowered unstimulated salivary pH (Lamy et al., 2011).

Many of the substances tested in our study influenced saliva flow, ensalivation, or ensalivation per unit of metabolic weight of cows. Overall, there was an augmentation in saliva flow rate to the rumen when the size of the ingested boli increased, as shown by the regression analysis. Capsaicin was responsible for the strongest effects on salivation dynamics. The low dose of capsaicin increased salivation rate and feed bolus size, suggesting that a low dose of this compound may promote the eating stimulus in ruminants. Conversely, the high dose reduced saliva flow, but increased feed ensalivation when expressed as g/g DM feed as well as per unit of metabolically active body tissue. Capsaicin is a compound that derives from chili peppers, and reacts with specific sensors, responsible for pain sensations (Caterina et al., 1997); this could have contributed to reduce feed intake with the high dosage. Gardner et al. (2020) found a similar increment in saliva flow rate after stimulation with capsaicin in humans. However, the effect was limited to the time that the subjects kept the treatment in the mouth and vanished soon after. Thus, the finding implies that capsaicin exerts a stimulatory effect on salivation when present in the mouth or during mastication. This effect would be particularly important in cows given the time they spend chewing the regurgitated digesta contents during rumination. Therefore, results from this study suggest the potential of a low dose of capsaicin to improve not only salivation, but also physico-chemical properties of saliva, and hence, a potential benefit on rumen pH regulation in cattle fed high-concentrate diets. Saliva flow rate to the rumen was also enhanced by ginger extract. Ginger is a root widely studied and known for its beneficial properties to the gastrointestinal tract (Ghayur and Gilani, 2005; Nikkhah Bodagh et al., 2019; Wang et al., 2020). Mardani et al. (2017) demonstrated a positive effect of ginger on saliva production in human patients affected by xerostomia. Our results indicate that ginger, possibly by the action of its active ingredients such as gingerol and shogaol, may display a similar effect in cattle. Given its cholinergic agonist-like activity, ginger probably stimulates the parasympathetic receptors in salivary glands to increase salivation (Ghayur and Gilani, 2005; Carpenter, 2013). The concomitant increase in the size of ingested boli also suggests an influence of the amount of ginger ingested on salivation stimulus.

L-menthol was another compound that tended to increase salivation in this study, and interestingly its low dose increased mean ruminal pH post feeding in our companion study (Castillo-Lopez et al., 2021b). Menthol has several isomers, and the L-(-) form has the strongest organoleptic properties and is the most common in nature (Eccles, 1994). Menthol is also the main component of mint essential oil. In our study, mint oil tended to increase salivation rate, and the low dose increased stimulated saliva phosphate. Mint flavor has been reported to increase saliva flow rate in humans (Davies et al., 2009). Specifically, *Mentha* species have been proven to inhibit acetylcholinesterase, an enzyme involved in the catabolism of neurotransmitters. This enzyme is present and active in bovine salivary glands, and its inhibition increases saliva flow rate (Liston et al., 2004; de Sousa Barros et al., 2015). Thus, results indicate that these phytogenic compounds may have a similar effect in cattle. It is suggested that menthol could have a direct effect on the olfactory-salivary reflex, given its strong aroma (Lee and Linden, 1992; Eccles, 1994; Haahr et al., 2004). However, the exact mechanism underlying the stimulation of saliva production by these compounds is not known yet, and in fact, the low dosage of mint oil reduced the ensalivation per unit of metabolic weight. Similarly, the tendency for the enhanced salivation with gentian root may be due to its organoleptic properties, and in particular to its bitterness. It is known that bitter taste stimulates specific neurons, that induce saliva flow (Matsuo et al., 2001; Travers and Geran, 2009; Mirzaee et al., 2017). In general, our findings for feed ensalivation are comparable with reported values in cattle (Beauchemin et al., 2008), but observations for salivation rate are lower compared to previous findings (Maekawa et al., 2002). Likely, differences in salivation may reflect dietary composition (Keesman et al., 2016), diet dry matter, animal age or animal physiological stage. For example, compared to our results, Chibisa et al. (2016) found greater flow rates of saliva with diets containing grater levels of dry matter, and using continental crossbred heifers as experimental unit.

The change in salivary osmolality when some of the compounds were supplemented may be due to their influence on ion channels. For example, angelica root contains components, such as ligustilide, that has been demonstrated to affect ion channels in other species (Huang et al., 2019). It is thus possible that this compound influenced the exchange channels in the oral cavity of the cows, disrupting the osmotic balance and consequently decreasing the ion content in saliva (Carr and Titchen, 1978). Nonetheless, long-term effects of the change in osmolality warrants further research.

## Conclusion

Phytogenic compounds tested in this study influenced salivation and salivary composition. Overall, most phytogenic compounds showed potential to enhance saliva flow or physico-chemical properties, and some of these effects were consistent with observed ruminal fermentation profile reported in the companion study. Thus, our results suggest the efficacy of these substances to contribute to animal health and to modulate ruminal fermentation in cattle fed concentrate-rich diets. Further research is needed to better understand and identify the mode of action of the phytogenic compounds. In particular, it would be important to investigate the mechanism of action of those compounds displaying an enhancement in salivary physico-chemical properties or dynamics. Given the dose-dependent responses observed for some of the compounds, it would also be essential to test a combination of these phytogenic compounds at specific doses, to determine any synergistic effects while avoiding undesired outcomes. Additionally, research is needed to evaluate long-term effects on health as well as milk production in dairy cattle fed high-concentrate diets. Our findings can provide an important starting point for further research to elucidate saliva dynamics in animals with high dry matter intake and a more complex metabolic status, such as high-producing dairy cows.

## Data Availability Statement

The original contributions presented in the study are included in the article/Supplementary Material, further inquiries can be directed to the corresponding author.

## Ethics Statement

The animal study was reviewed and approved by the Institutional Ethics and Animal Welfare Committee of the University of Veterinary Medicine Vienna and the Austrian National Authority.

## Author Contributions

QZ acquired funding and led the CD laboratory. QZ, RP, and NR designed the experiment, that was performed by SR, RR-C, RP, and EC-L. AS-A and SS performed the lab analysis. EC-L analyzed the data. SR and RR-C wrote the manuscript, that was read and approved by all authors.

## Conflict of Interest

NR was employed by the company BIOMIN Holding GmbH. The authors declare that this study received funding from BIOMIN Holding GmbH. The funder had the following involvement with the study: processed and provided phytogenic compounds that were evaluated. The funder was not involved in collection, analysis, or interpretation of data.
