# Supplementary material for: Supplementation With Phytogenic Compounds Modulates Salivation and Salivary Physico-Chemical Composition in Cattle Fed a High-Concentrate Diet
Source: Front Physiol. 2021 Jun 3;12:645529. doi: 10.3389/fphys.2021.645529 (PMC8209472; doi:10.3389/fphys.2021.645529)
Supplement: Supplementary file 1 [file Data_Sheet_1.docx]

Supplementary Material

# Supplementary Tables

**Table S1**. Ingredients, chemical composition, and physically effective NDF (peNDF) of the total mixed ration (TMR) fed to the cows, which was used to prepare the controlled meal.

| **Ingredients** | **% of DM** |
| --- | --- |
| Grass silage | 26.25 |
| Corn silage | 8.75 |
| Rolled wheat grain | 26.00 |
| Pelleted concentrate mixture^1^ | 39.00 |
| **TMR chemical composition** | **% of DM (unless otherwise stated)** |
| DM, % as fresh | 46.4 ± 0.84 |
| Crude protein (CP) | 16.5 ± 1.41 |
| Neutral detergent fiber (NDF) | 32.4 ± 1.33 |
| Acid detergent fiber (ADF) | 19.7 ± 1.21 |
| Starch | 32.8 ± 1.75 |
| Ether extract | 3.0 ± 0.13 |
| Non-fiber carbohydrates | 41.9 ± 1.72 |
| Ash | 6.2 ± 0.10 |
| peNDF^2^ >8 mm | 12.9 ± 0.6 |

^1^The pelleted concentrate mixture contained: triticale (3.75%), wheat (11.25%), rapeseed meal (34.0%), bakery by-product (45.0%), molasses (3.0%), mineral-vitamin premix for dairy cattle (2.0%), and limestone (1.0%).

^2^Physically effective NDF.

**Table S2.** Substances tested, scientific name of the plant source and relative dosages. Each substance (in powder) was mixed in 2.5 kg (dry matter basis) of feed.

| Substance | Scientific name of plant source | 1x dosage (mg/kg) | 10x dosage (mg/kg) |
| --- | --- | --- | --- |
| Angelica root | *Angelica archangelica* L. | 6.6 | 66 |
| Capsaicin | *Capsicum* sp. | 10 | 100 |
| Garlic oil | *Allium sativum* L. | 0.3 | 3 |
| Gentian root | *Gentiana lutea* L. | 6.6 | 66 |
| Ginger | *Zingiber officinale* | 40 | 400 |
| L-menthol | *Mentha arvensis* L. | 6.7 | 67 |
| Mint oil | *Mentha arvensis* L. | 15.3 | 153 |
| Thyme oil | *Thymus vulgaris* L. and *Thymus zygis* L. | 9.4 | 94 |
| Thymol | Chemical synthesis | 5 | 50 |

**Table S3.** Effect of supplementation with angelica root on salivary physico-chemical properties, salivation and feed bolus dynamics of non-lactating Holstein dairy cows.

|  | Treatment^1^ | | |  | *P*-value | |
| --- | --- | --- | --- | --- | --- | --- |
| Unstimulated saliva^2^ | CON | LOW | HIGH | SEM^4^ | Linear | Quadratic |
| pH | 8.81 | 8.73 | 8.76 | 0.067 | 0.61 | 0.52 |
| Bicarbonate, mM | 77.09 | 90.00 | 80.75 | 8.918 | 0.64 | 0.17 |
| Phosphate, mM | 10.54 | 9.95 | 11.67 | 1.117 | 0.42 | 0.35 |
| Total proteins, µg/mL | 312.8 | 370.0 | 283.0 | 42.47 | 0.58 | 0.19 |
| Buffer capacity, mol of HCl/L/ΔpH | 0.014 | 0.015 | 0.014 | 0.0009 | 0.99 | 0.60 |
| Osmolality, mOsm/kg | 254.6 | 241.9 | 249.7 | 3.18 | 0.05 | <0.01 |
| Lysozyme activity, U/mL/min | 38.58 | 30.11 | 36.89 | 8.306 | 0.85 | 0.39 |
| Mucins, mg/mL | 1.55 | 1.37 | 1.44 | 0.318 | 0.62 | 0.56 |
| Stimulated saliva^3^ |  |  |  |  |  |  |
| pH | 6.81 | 6.61 | 6.69 | 0.069 | 0.17 | 0.09 |
| Phosphate, mM | 12.60 | 12.99 | 13.58 | 1.182 | 0.48 | 0.94 |
| Buffer capacity, mol of HCl/L/ΔpH | 0.038 | 0.033 | 0.035 | 0.0033 | 0.32 | 0.30 |
| Osmolality, mOsmol/kg | 386.0 | 326.8 | 393.6 | 64.10 | 0.82 | 0.07 |
| Saliva dynamics |  |  |  |  |  |  |
| Salivation rate, g/min | 74.32 | 94.99 | 81.48 | 10.477 | 0.55 | 0.17 |
| Ensalivation, g/g DM feed | 4.18 | 4.34 | 4.25 | 0.356 | 0.86 | 0.75 |
| Ensalivation, l/kg DM feed/kg LW^0.75^ | 0.883 | 1.198 | 1.015 | 0.123 | 0.33 | 0.08 |
| Bolus size (as is), g | 223.54 | 280.56 | 249.79 | 31.226 | 0.47 | 0.23 |
| Bolus size (DM), g | 37.52 | 46.47 | 43.18 | 6.745 | 0.47 | 0.44 |

^1^CON: a control diet prepared by combining 50 g of the wheat silica carrier and 2.5 kg (DM) of TMR; LOW: 6.6 ppm of angelica root in 2.5 kg (DM) of TMR; HIGH: 66 ppm of angelica root in 2.5 kg (DM) of TMR.

^2^Saliva samples collected from the mouth after treatment intake; data from samples collected before treatment were used as covariates.

^3^Saliva samples collected at cardia by collecting and straining saliva from ingested feed boli.

^4^The largest standard error of the mean.

**Table S4.** Effect of supplementation with gentian root on salivary physico-chemical properties, salivation and feed bolus dynamics of non-lactating Holstein dairy cows.

|  | Treatment^1^ | | |  | *P*-value | |
| --- | --- | --- | --- | --- | --- | --- |
| Unstimulated saliva^2^ | CON | LOW | HIGH | SEM^4^ | Linear | Quadratic |
| pH | 8.87 | 8.80 | 8.70 | 0.052 | 0.03 | 0.84 |
| Bicarbonate, mM | 74.53 | 88.67 | 85.58 | 9.142 | 0.33 | 0.40 |
| Phosphate, mM | 9.21 | 9.39 | 9.30 | 1.580 | 0.96 | 0.93 |
| Total proteins, µg/mL | 315.8 | 226.1 | 163.4 | 82.65 | 0.10 | 0.84 |
| Buffer capacity, mol of HCl/L/ΔpH | 0.013 | 0.013 | 0.014 | 0.0011 | 0.93 | 0.82 |
| Osmolality, mOsm/kg | 250.1 | 246.4 | 249.6 | 4.38 | 0.88 | 0.26 |
| Lysozyme activity, U/mL/min | 41.64 | 41.12 | 32.18 | 9.078 | 0.40 | 0.69 |
| Mucins, mg/mL | 1.73 | 1.61 | 2.00 | 0.824 | 0.84 | 0.79 |
| Stimulated saliva^3^ |  |  |  |  |  |  |
| pH | 6.85 | 6.62 | 6.69 | 0.095 | 0.06 | 0.02 |
| Phosphate, mM | 11.41 | 14.10 | 10.81 | 1.969 | 0.70 | 0.02 |
| Buffer capacity, mol of HCl/L/ΔpH | 0.039 | 0.084 | 0.045 | 0.0270 | 0.82 | 0.05 |
| Osmolality, mOsmol/kg | 436.0 | 455.4 | 428.4 | 40.97 | 0.81 | 0.36 |
| Saliva dynamics |  |  |  |  |  |  |
| Salivation rate, g/min | 75.16 | 85.00 | 90.24 | 7.338 | 0.08 | 0.74 |
| Ensalivation, g/g DM feed | 4.54 | 4.51 | 4.14 | 0.733 | 0.53 | 0.76 |
| Ensalivation, l/kg DM feed/kg LW^0.75^ | 0.062 | 0.060 | 0.051 | 0.014 | 0.42 | 0.75 |
| Bolus size (as is), g | 231.20 | 258.35 | 288.78 | 31.27 | 0.08 | 0.95 |
| Bolus size (DM), g | 36.80 | 43.18 | 48.80 | 8.347 | 0.11 | 0.95 |

^1^CON: a control diet prepared by combining 50 g of the wheat silica carrier and 2.5 kg (DM) of TMR; LOW: 6.6 ppm of gentian root in 2.5 kg (DM) of TMR; HIGH: 66 ppm of gentian root in 2.5 kg (DM) of TMR.

^2^Saliva samples collected from the mouth after treatment intake; data from samples collected before treatment were used as covariates.

^3^Saliva samples collected at cardia, by collecting and straining saliva from ingested feed boli.

^4^The largest standard error of the mean.
